# An undue emphasis on rural older adults in the Chief Medical Officer's annual report 2023?

**DOI:** 10.1016/j.clinme.2024.100204

**Published:** 2024-04-23

**Authors:** Nathan J. Cheetham, Jugdeep Dhesi, Adrian Hopper, Jennifer B. Dowd, Claire J. Steves

**Affiliations:** a Department of Twin Research and Genetic Epidemiology, King's College London, London, United Kingdom; b Guy's & St Thomas's NHS Foundation Trust, London, United Kingdom; c Leverhulme Centre for Demographic Science, Nuffield Department of Population Health, University of Oxford, Oxford, United Kingdom

**Keywords:** Health policy, Ageing, Regional inequality

## Abstract

The Chief Medical Officer's annual report 2023 presents an incomplete and skewed picture of the geography of older people in England. We show that there are higher absolute numbers of older people in urban areas in England and Wales, in contrast to key messages from the CMO report which suggest greater need in rural areas based on relative metrics. The absolute size of the urban–rural difference in the population of older people is projected to grow by 2043. Older adults in urban areas are much more likely to live in deprived areas than older adults in rural areas. The absolute number and prevalence of older adults in poorer health is also higher in urban areas, leading to greater healthcare needs. Policy-makers need to consider both absolute and relative demographic trends as well as making use of direct measures of health when planning how healthcare services for older adults are distributed geographically in England.

## Spotlighting health and geography of older adults

The latest annual report from the Chief Medical Officer (CMO) for England,^1^ shone a timely spotlight on older people's health, emphasising the need for policy makers to understand the geography of ageing in England to best plan and allocate resources. Key passages of the Foreword and Executive Summary and the official press release^2^ focused on older people in rural and coastal areas. However, the conclusions, quoted below, appeared to draw solely on variation in the *relative* population share of older adults across local areas. Here, we show that examining the *absolute* numbers of older adults paints a very different picture.

The report states:

*‘The great majority of people move out of cities and large towns before older age,****concentrating geographically in coastal, semi-rural or peripheral areas****….’ (CMO report, foreword, page 2)*

*‘…resources should be directed towards areas of greatest need,****which include peripheral, rural and coastal regions of the country’.****(CMO report, recommendations, page 9)*

*‘The****geography of older age in England is already skewed away from large urban areas towards more rural, coastal and other peripheral areas****, and will become more so.’ (GOV.UK press release)*

**Fig. 1:**
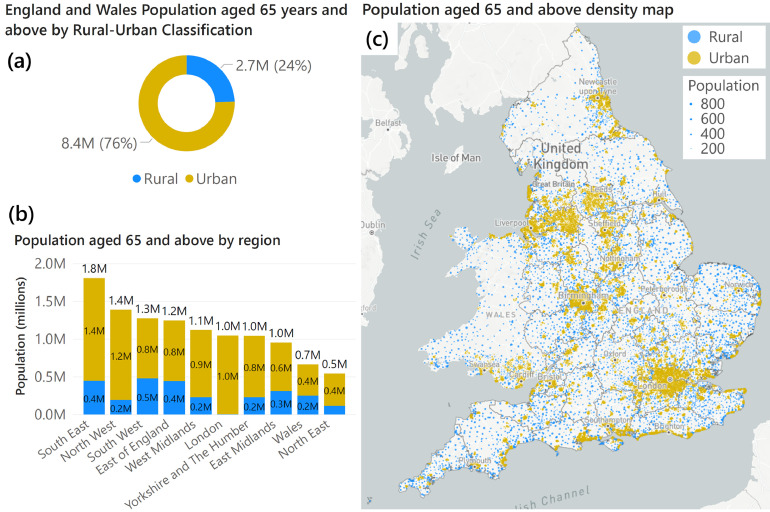
(a) Population aged 65 years or above in England and Wales, split by the rural–urban classification of the lower super output area (LSOA). (b) Regional population aged 65 years or above split by rural–urban classification. (c) Population aged 65 years or above in England and Wales as a population dot density map. Density map generated from LSOA level population sizes. Dots are located at the centre of each LSOA, and dot areas are scaled proportionally to population. Dots are coloured by the Rural–Urban classification of the respective LSOA. NHS Integrated Care Board boundaries (April 2022) in England are also displayed for reference. Data from Census 2021 LSOA level population by age figures^3^, linked to the 2011 Rural–Urban Classification of LSOAs (binary version).^4^

From recent Census 2021 data, we see that most older people (most often defined in the CMO report as aged 65 years and above) do not live in rural areas (Fig. 1). Similar to the overall population in England and Wales, where 82% (48.8 million of 59.6 million) live in urban areas, three in four older people live in urban areas in England and Wales (8.4 million of 11.1 million, 76%). Furthermore, all 10 United Kingdom (UK) regions have a large urban-dwelling majority of older adults.

Maps shown on the front cover of the CMO 2023 report presenting the *proportion* of older adults aged 75 and above by local area should not be misinterpreted as showing *where* most older people live in *absolute* numbers. While relative proportion maps could be interpreted as showing ‘coldspots’ or absences of older adults in urban areas including Greater Manchester, London, Merseyside, and West Yorkshire, mapping *absolute* population shows how older adults are in fact concentrated in urban areas.

## Urban versus rural population growth

Moreover, the CMO report states:

*‘****Urban areas are not where the growth in older people is occurring****; more peripheral areas are where the increase in need will be seen. In planning health and social care services, as well as infrastructure, this report makes clear that the****geography of older age in the UK is already highly skewed away from large urban areas, and will become more so****.’ (CMO report, executive summary, page 3)*

**Fig. 2:**
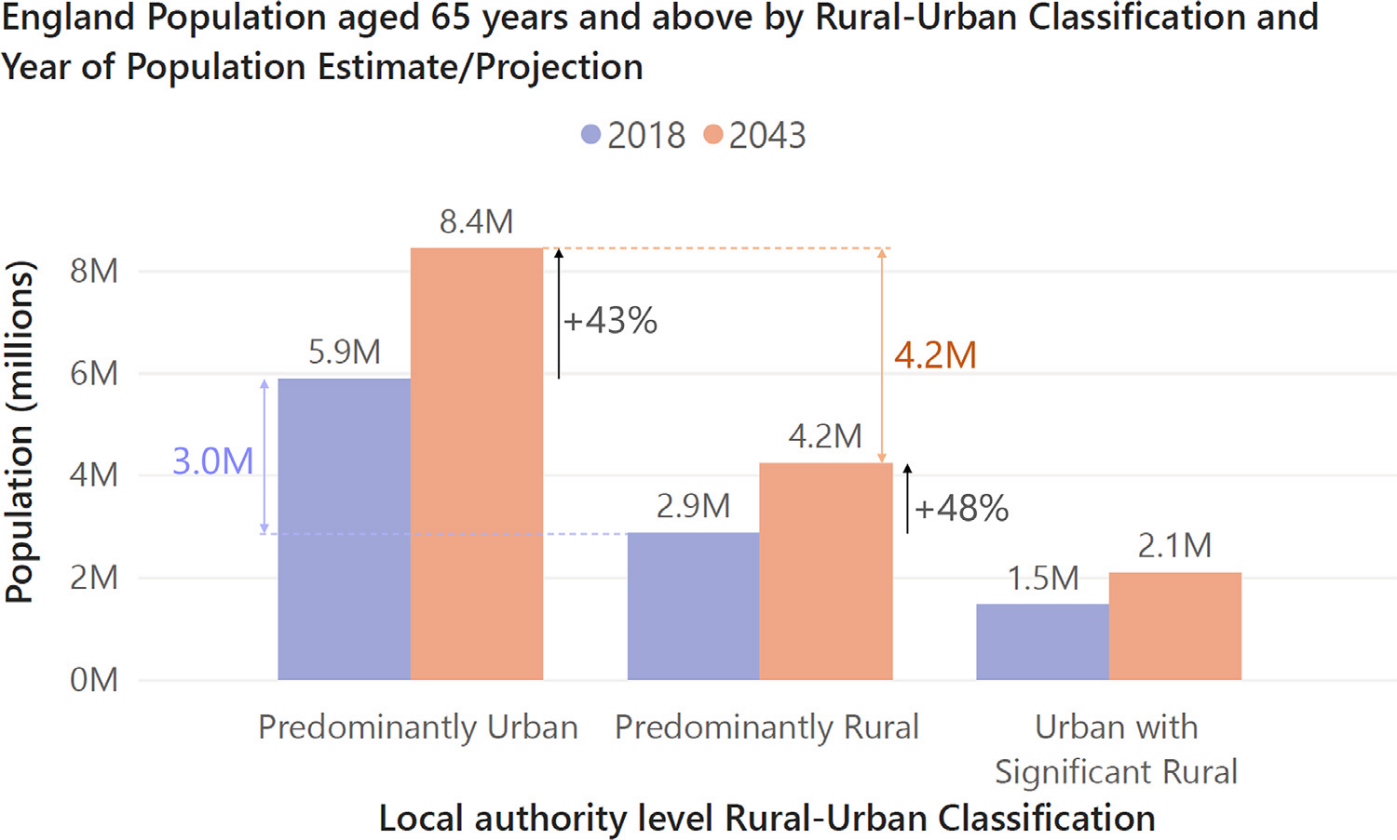
Population aged 65 years or above in 2018 (estimated) and 2043 (projected), split by the rural–urban classification of their local authority. Data from latest subnational population projections based on mid-2018 population estimates (as was also used in the CMO 2023 report),^5^ linked with the 2011 Rural–Urban classification of local authority areas (3-fold classification). Note: data includes England only.

However, population growth of urban-dwelling older adults is actually projected to be greater than for rural-dwelling older adults in England over the next 20 years, as shown in Fig. 2.^5^ Relative population growth is only slightly smaller in urban areas at +43%, in comparison to +48% in rural areas. By 2043, there are projected to be 4.2 million more older adults living in predominantly urban than predominantly rural local authority areas, versus the 3.0 million difference at the original mid-2018 estimates used to generate the projections.

## Understanding area-specific issues

This said, we do not wish to minimise issues faced in rural areas and welcome important points made in the CMO report. Critically, the older age to working age population ratio is higher in rural areas than urban areas, and the ratio is projected to increase over time. This may create pressures in staffing healthcare services in rural areas, where there may be smaller local pools to draw staff from, in combination with higher healthcare needs associated with a larger population share of older adults. Other issues include larger distances between patient and care and lower digital connectivity.^6^

**Fig. 3:**
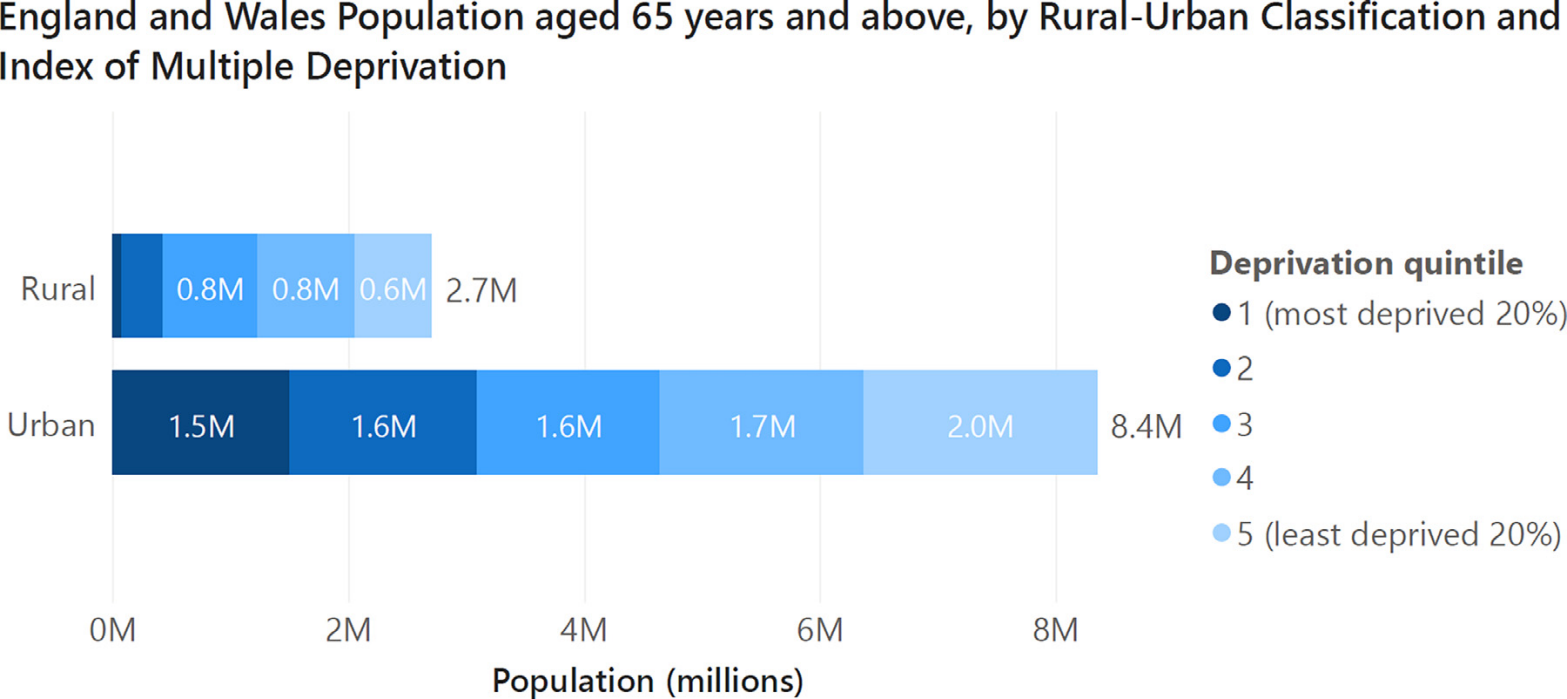
Population aged 65 years or above split by the Index of Multiple deprivation rank quintile and rural–urban classification of lower super output areas. Data from Census 2021 lower super output area level population by age figures,^3^ linked to the 2011 Rural–Urban Classification of lower super output areas (binary version)^4^ and 2019 versions of the index of multiple deprivation for England^9^ and Wales.^10^

Balanced against specific challenges in rural areas are issues of social inequality between and within neighbourhoods, given the strong influence of social determinants on healthy ageing.^7^ This is likely exacerbated by reduced healthcare provision, lower funding and lower quality ratings in primary care in more deprived areas.^8^ With this in mind, we highlight that as well as being larger in absolute number, urban-dwelling older adults also tend to live in more deprived neighbourhoods than their rural-dwelling counterparts. To illustrate this point, only 3% of rural-dwelling over 65s currently live in the most deprived 20% of areas in England of Wales, in comparison to 18% of urban-dwelling older adults. In absolute terms, as shown in Fig. 3, there are more older adults living in the 40% most deprived local areas (3.1 million) than there are in all rural areas in England and Wales (2.7 million).

## Measuring health directly

**Fig. 4:**
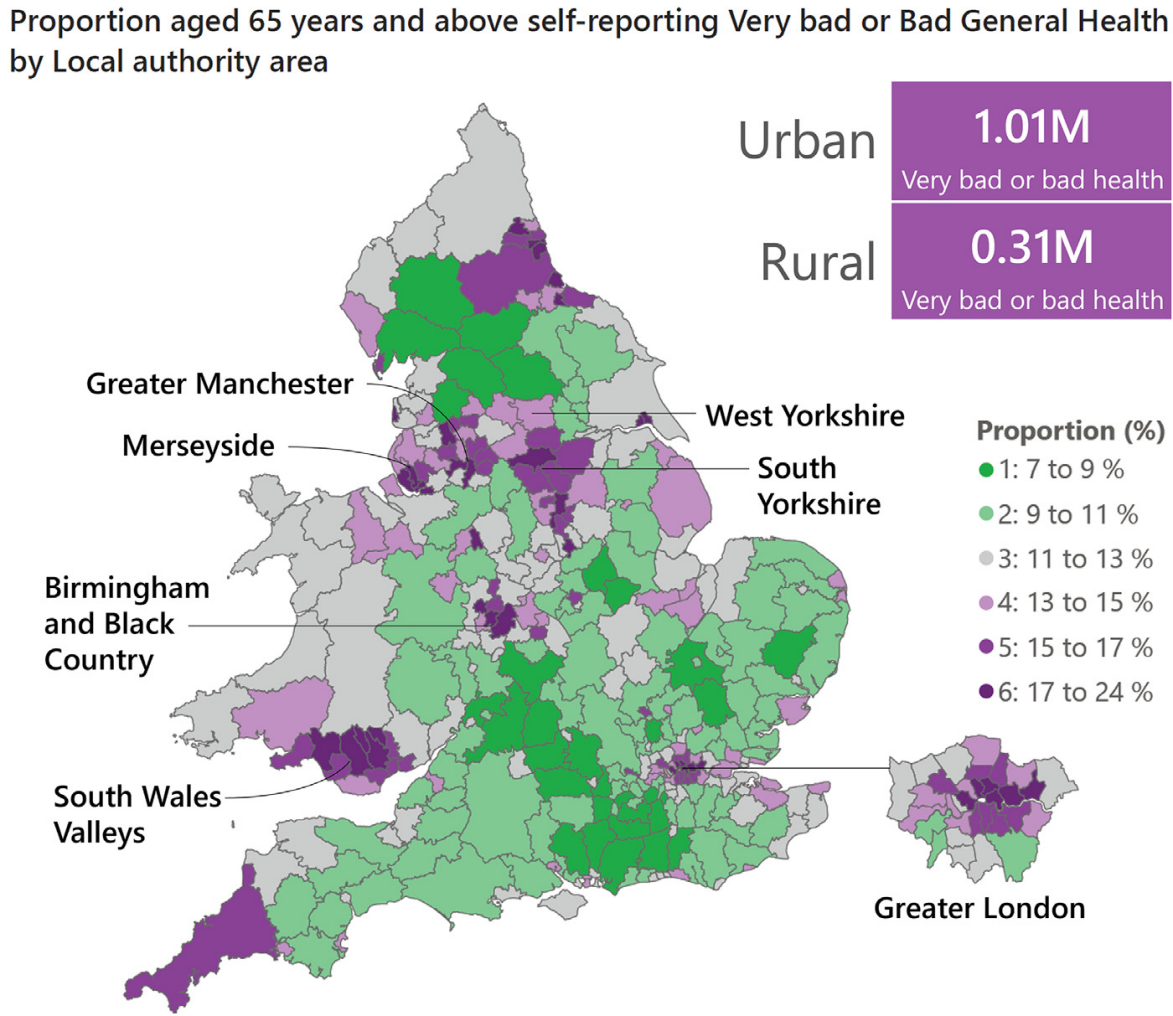
Choropleth map of proportion of population aged 65 years or above in England and Wales self-reporting very bad or bad general health in the 2021 Census by local authority area. Boxes show population sizes self-reporting very bad or bad health living in urban (combining predominantly urban with urban and significant rural) and predominantly rural local authority areas. Data from Census 2021 local authority area level general health by age figures,^14^ linked with the 2011 Rural–Urban classification of local authority areas (3-fold classification).

As well as looking at proxies for healthcare need such as age and deprivation, we can look directly at how older people rated their own general health in the 2021 Census, a single-question measure consistently linked to healthcare service use^11^ and future mortality.^12^ As presented in Fig. 4, we find that urban local authority areas have higher absolute numbers and tend to have higher proportions of older people reporting their health as ‘very bad’ or ‘bad’ in comparison to those living in rural areas. The geographic distribution of older adults in poor health echoes on a representative, national scale, a recent report by Sinclair *et al* (highlighted in the CMO report Fig. 5.4) that showed hotspots of frailty in urban and coastal areas within a much smaller sample.^13^

Using absolute and relative statistics *in combination* can help identify areas that are particularly vulnerable. For example, older adults are high in both absolute number and relative population share in Cornwall and Dorset. Meanwhile, in Birmingham and Black Country, West Yorkshire, South Yorkshire and Cheshire and Merseyside, older adults are high in absolute number and have a relatively high prevalence of bad health.

## Going forwards

Looking to the future, the growing effects of global heating over time may exacerbate existing urban-rural inequalities in healthy ageing. Older people are at increased risk during periods of heat stress,^15^ and differences in housing stock and heat island effects are likely to lead to disproportionate effects in urban areas,^16^ as seen in the 2022 UK heatwaves. Likewise, novel infections, such as COVID-19 hit urban areas of higher deprivation first and hardest,^17^ where older people again were most susceptible.

Overall, we caution against misinterpretation of the headlines from the CMO annual report based on relative metrics. There should instead be a focus on the distribution of the absolute number of older people, alongside direct measures of health and the social circumstances that contribute to health, when allocating workforce and infrastructure resources for healthcare services. Finally, we welcome the report's focus on developing resilience in future ageing populations. Those younger now will later be older, and so investment in preventive measures that promote healthy ageing is needed across all ages, and in all areas.

## Declaration of competing interest

CJS declares personal factors which might be perceived as a conflict with regard to workforce planning for older people. She works as a geriatrician in an urban area, but this potential conflict is balanced by the fact that she has close relatives aged over 75 living in rural and coastal areas.
